# Workplace Interactional Demands and Work-Family Enrichment: An Investigation From the Service Sector

**DOI:** 10.3389/fpsyg.2020.01476

**Published:** 2020-06-26

**Authors:** Saira Solat, Muhammad Abrar, Rizwan Shabbir, Mohsin Bashir, Sharjeel Saleem, Shahnawaz Saqib

**Affiliations:** Lyallpur Business School, Government College University Faisalabad, Faisalabad, Pakistan

**Keywords:** workplace interactional demands, work-family enrichment, personal accomplishment, relatedness, emotional labor

## Abstract

Emotional labor has, so far, been found to have negative consequences for service sector employees’ personal well-being. This study strives to look at the positive aspect of emotional labor in the interactive jobs. This research focuses on employees’ psychological needs fulfillment through workplace interactions. The current research is an effort to highlight the importance of workplace interactions through fulfilling the employees’ need for relatedness and personal accomplishment, which triggers positive resources that can be transferred from work domain to home domain and might induce work-family enrichment. Primary data were gathered through self-administered questionnaires. The target population was nurses working in public and private hospitals located in Faisalabad, Lahore, and Multan cities of the province of Punjab, Pakistan. This sector needs the attention of the policymakers if we want to enhance the performance of health sector employees and to improve their work-home enrichment as a healthy worker is a productive worker. Demographic characteristics of respondents have been identified through SPSS while Structural Equation Modeling (SEM) was used as multivariate data analysis tool for statistical analysis. Assessment of measurement and structural model was found satisfactory. The results showed that workplace interactional demands had a significant positive effect on work-family enrichment. Moreover, relatedness and personal accomplishment partially mediated the relationship between workplace interactional demands and work-family enrichment. The findings of the study revealed that workplace interactions are critical in fulfilling the basic psychological needs of individuals and as a result, they find themselves energized and achieve their full potential through the fulfillment of their need for relatedness and accomplishment. This energy is a valuable resource that can enrich their family life.

## Introduction

The service-oriented economy has globally transformed the service structure of the organizations by enhancing the efficiency and effectiveness of people’s work (Erickson and Ritter, 2001). As members of a social community, people perform beyond their work roles that highlighted the importance of work-family enrichment aspects (Halbesleben, 2011). The employees who are working in an interactive environment might experience work-family enrichment as explored by Bhave and Lefter (2018), which highlighted the importance of workplace interactions in work-family enrichment through vitality. As a downside, workplace interactions result in fatigue at work which causes deterioration of life at home due to resource depletion (Voydanoff, 1987).

Specifically, in the service sector, interactive jobs engage employees in emotional labor because they hide their genuine emotions to display positive emotions and transfer work fatigue to home-domain activities (Grandey, 2000; Brotheridge and Grandey, 2002). The established assumptions about emotional labor concluded that workplace interactional demands/occupational interaction requirements are resource depleting (resource draining), which results in service-providers experiencing burnout (Grandey et al., 2015). However, this prevailing assumption has been challenged through several ideas that are pitched from different perspectives of work recovery with a focus on the respites and breaks to recover valuable resources (Beal et al., 2005) and work design by introducing relational job design to spark employee motivation (Grant, 2007).

The bright side of emotional labor highlighted that harmful effects of workplace interactional demands could not be attributed to the nature of interactive jobs; instead, these deleterious outcomes arise because of use of poor tactics for emotion regulation as well as maladapted person-job fit, poor working conditions, misrecognition of employee needs by management and a lack of autonomy given to the employees (e.g., Grandey et al., 2013; Bhave and Lefter, 2018). Thus, if strategies are adequately adopted by service organizations, emotional labor can be constructive for employees as well as for organizations (Humphrey et al., 2015).

The work of Grandey et al. (2013) suggested three categories related to interactional demands from emotional labor perspective, which are workplace interactional demands, emotional presentation/behavioral expression of emotions, and intrapsychic processes/subjective emotional control strategies enforced to manage emotions. This study adopted workplace interactional demands by exploring the nursing profession as they interact with the beneficiaries of their work, i.e., patients. The interactional demands result in gaining social resources that enhance employees’ energy-level by fulfilling the need for relatedness and personal accomplishment. These energy resources could be transferred from work-domain to home-domain (ten Brummelhuis and Bakker, 2012) and impact family enrichment (Greenhaus and Powell, 2006). Furthermore, workplace interactions are related positively to job satisfaction (Bhave and Glomb, 2016) and feelings of personal accomplishment (Brotheridge and Grandey, 2002).

The study of Greenhaus and Powell (2006) defined work-family enrichment as the extent to which experiences in one role improve the quality of life in the other role. It can be achieved by fulfilling the employees’ need for relatedness and accomplishment. The interactions that have replenishing capabilities mainly serve as an adjusting break with transitory effects upon individuals who are caregivers (nursing staff, etc.), and these interactions also have the successive ability of self-regulation. The resources gained in the performance of one role can serve as an essential reservoir for performing other roles, so resources gained at work might result in family enrichment (Rothbard, 2001; ten Brummelhuis and Bakker, 2012). The work, if properly managed, can serve as recovery for employees, particularly when they interact with the beneficiaries of their work; in the nursing profession, for instance, the caregivers have interactions with their beneficiaries (patients).

Therefore, emotional labor, as occupational interactional requirements/workplace interactional demands, is resource replenishing rather than resource-draining (Bhave and Lefter, 2018). Firstly, there are several studies that highlight that interactional demands can result in the fulfillment of basic human need for relatedness (Alderfer, 1972). These interactions enhance positive mood (Watson, 2000a) and well-being (Cohen and Wills, 1985; Deci et al., 2001; Warr, 2007). Secondly, the work design literature stated that workplace interactions have a motivating effect on employees’ well-being (Grant, 2007) and positive mood (Watson, 2000b), which derive work-family enrichment. Lastly, interactional demands for caring professions that carry with them a high degree of altruism like nursing have restorative potential which is essential for employees’ well-being and could have a favorable impact on their family life.

The contributions of this study address several aspects. Firstly, it proposes that the positive side of interactional demands can play a vital role in understanding and designing the activities for employee recovery because traditionally employees serving in highly interactional occupations that involve frequent interactions with others are thought to be exhausted at work. Therefore, they require a greater need for recovery (Lilius, 2012; Zhang, 2019). Prior studies on employee recovery have emphasized the importance of breaks and incorporation of leisure activities in work to gear up employee recovery (Sonnentag and Fritz, 2007; Trougakos and Hideg, 2009). However, the current research suggests that work itself can be an essential source of employee recovery which is needed for the nursing staff as due to long work hours, they have less time for leisure. This perspective has significant implications on how the work should be designed and how the recovery of employees is possible at work and home domains.

Secondly, enrichment in terms of energy resources in one domain can serve as fuel for enrichment in the other domains. So, the issue regarding employee engagement needs to be addressed because if the employee is in the affirmative and active psychological state, s/he can work with more dedication, vigor, and absorption (Bakker et al., 2016). Thus, it has important managerial/practical implications in the field of organizational health services (Bakker et al., 2008; Schaufeli and Bakker, 2010).

Thirdly, the current study contributes to the empirical literature on emotional labor and proves that workplace interactional demands contribute directly to work-family enrichment, which is a blessing for the service industry. Bhave and Lefter (2018) investigated the indirect effects of interactional demands on work-family enrichment and called for future researchers to check the direct effects as well. They encouraged future researchers to investigate alternate paths. They suggested emphasizing the occupations that are typified by values of high altruism (“foster harmony and service to others”; e.g., nurses and childcare workers). This study attempts to not only check the direct effect but also attempts to explore the mediating role of relatedness and personal accomplishment in the relationship between interactional demands and work-family enrichment as relatedness is the human need that can be coupled with interpersonal interactions (Alderfer, 1972).

Lastly, the nursing sector in Pakistan needs the attention of policymakers and researchers as it is characterized by long working hours, workplace violence, low compensation, and, most of all, they are exposed to health hazards. The nursing staff is overburdened in the Pakistani healthcare sector. Approximately 99,228 nurses are registered all over Pakistan (Pakistan Economic Survey, 2017), and the population per nurse ratio is 1/2200 individuals. Quality of healthcare services could hamper significantly when nurses failed to achieve work-family enrichment under a great deal of burden. So, this research is quite helpful in the enrichment of their family lives. If they feel energized, they can perform their duties more efficiently. Moreover, especially women professionals were not studied separately; most of the previous research laid emphasis upon the family roles that include spouse and parents irrespective of gender. So, there is a need to study this aspect in developing countries like Pakistan. Bakker et al. (2011) summarized that women have to play the roles of family members and their work-family enrichment should be enhanced. According to International Labor Organization, globally female participation in the labor force has been doubled to 24.10% in 2019 from 12.51% in 1995.

The research objective of the current study is to examine the bright side of the workplace interactional demands. Moreover, we attempt to study the role of workplace interactional demands toward enhancing work-family enrichment for service sector employees through the underlying mechanism of relatedness and personal accomplishment.

### Theoretical Foundation and Hypotheses Development

The hypotheses in the study are based on the notions of self-determination theory (SDT) and the conservation of resources (COR) theory. SDT is a macro theory of human motivation that focuses on the fulfillment of basic psychological needs of relatedness, competence, and autonomy. In the context of current research, the nurses, when interacting with the patients, fulfill their need for relatedness and belongingness. This results in the gain of resources that can be conserved by the nurses and can be transferred from the work domain to the home domain. The COR theory states that employees gain as well as conserve resources that can be transferred from the work domain to the home domain and can result in work-family enrichment. The healthcare organizations and hospital administration look for better ways to protect their nursing staff from resource drainage. In this context, the current research emphasizes the fulfillment of the need for relatedness through nurse-patient interaction, which might result in resource gain that is useful for the home domain as well.

### Workplace Interactional Demands and Work-Family Enrichment

According to SDT (Ryan and Deci, 2000), the fulfillment of basic needs gives energy to the employees which could travel from the work domain to the home domain. The prior research on work design contends that workplace interactions, especially those with the beneficiaries of work, result in employee motivation and enhance their well-being as well (Fried et al., 2007). However, another stream of recovery literature contends that the breaks during work have restorative potential (Lilius, 2012). Prior studies on emotional labor have mixed findings on the subject that workplace interactions themselves are a source of employee burnout. Wharton (2009) has highlighted the positive side of workplace interactions. We might interpret that workplace interactions have restorative properties. If the above proposition is true, then the critical question that needs clarification is that if workplace interactions are resource replenishing/energizing, can it be transferred from one domain to another domain? In other words, can workplace interactions result in work-home enrichment? Or to put it in simple words how this energy is channeled from work domain to home domain? The above questions could also be answered through the propositions of the COR theory (Hobfoll, 1989; Halbesleben et al., 2014). This theory states that workplace interactions are background social resources that can generate personal resources of relatedness, these resources energize the employees and this energy resource enriches their family life.

Greenhaus and Powell (2006) proposed the theory of work-family enrichment on the basis of the notion that positive effects can transit amongst work and family life. The above theory states that work experiences have the capabilities to enrich family life and vice versa. For example, the interpersonal skills that the people learn at work can serve as an essential resource to enhance the quality of family life, or we can say that communication skills that parents learn during their course of work improve their interactions with children and other family members. Work-family enrichment could be explained from the perspective of role accumulation theory developed by Sieber (1974). This theory states that both psychological, as well as tangible resources, are generated through engagement in multiple high-quality roles. Prior research conducted by Barnett and Hyde (2001) states that there is a positive association between well-being and role accumulation.

Previous studies have examined the relationship between workplace interactional demands and work-family enrichment. For instance, Liu et al. (2018) examined the relationship between deep acting emotional labor and family quality through the mediation of deep acting at home. Wu et al. (2020) found deep acting emotional labor positively related to work-family enrichment. Keeping in view the propositions of work-family enrichment theory, we propose the following hypothesis:

***H1:*** *There is a positive association between workplace interactional demands and work-family enrichment.*

### Workplace Interactional Demands, Relatedness and Work-Family Enrichment

SDT provides a comprehensive foundation or skeleton that supports the human motivation as well as personality traits that emphasize human’s inherent propensities toward self-actualization through the satisfaction of their basic psychological needs for autonomy, competence, and relatedness (Ryan and Deci, 2017). This theory is often used to identify the universal characteristics of human nature as well as individual differences in order to map out ephemeral involvements as well as substantial cultural and social processes. Furthermore, the visions of SDT are universally applied in nearly all vital domains of life, e.g., work, education, psychotherapy, and the likes. The satisfaction of psychological needs is a predictor of well-being and intrinsic motivation across cultures in all life domains. The core proposition of SDT is that human beings naturally attain their full potential under circumstances that facilitate the satisfaction of their basic psychological needs. Every individual has three basic needs i.e., “need for autonomy, need for competence, and need for relatedness.” When these needs of individuals are satisfied, then they arrive in a self-directed mode which enhances their well-being by fostering intrinsic motivation. One of the psychological needs that are inherent in human nature is relatedness, or in other words, the need to feel cherished and connected with others. The satisfaction of these basic needs enhances the energy levels of employees. This is particularly important in the context of the nursing sector as they must stay motivated and energized to give their best as they are involved in patient care and life-saving concerns.

As COR theory describes the motivation, it looks practical to check other theories of motivation to clarify further the mechanisms under which a person possibly places value upon resources. Human beings have to perform multiple roles, and they need resource base to perform those roles actively. As per SDT, there exists a continuum of motivation starting from amotivation and ending on intrinsic motivation, and in between them come multiple stages of extrinsic motivation (Gagné and Deci, 2005). The satisfaction of basic human needs for autonomy, competence and relatedness enhances progression along that continuum (Ryan and Deci, 2000). When basic human needs are met, the individuals feel greater heights of well-being (Chirkov et al., 2003; Deci and Ryan, 2008). Similar propositions are also given by Deci et al. (2001), Kovjanic et al. (2013), and Vandercammen et al. (2014) regarding fulfillment of basic psychological needs which predict employee motivation as well as on-the-job psychological adjustment.

The fulfillment of basic needs can be attributed to enhanced energy levels of workers. In order to address the concern of channelizing this energy from one domain to another domain, we look at the notions of the COR theory. Drawing on the COR theory and SDT, we can set the foundations of our research. These concepts are quite helpful in understanding the value of resources and explain why individuals need to acquire and conserve resources.

It is quite interesting to observe that all three basic needs that are mentioned in SDT are examined mainly against the backdrop of the COR theory, especially while considering social relations in meeting the need for relatedness. So it can be stated that workplace interactions help in the fulfillment of the basic psychological needs of employees and as a result, their energy levels are elevated and they gain important resources. The COR theory states that these resources can be conserved by the employees and can be utilized in the home domain that might enrich their family lives. Resources gained during work can be transferred to the home domain (Halbesleben et al., 2014). When we talk about the nursing sector, this is particularly important in third world countries like Pakistan, where they have to work for longer hours and must carry energy to the family domain.

Research by ten Brummelhuis and Bakker (2012) has stated how resources gained at work can become useful resources for the family domain. This emerging stream of work-family research seems to pinpoint that how performing work roles can enhance the performance of family roles and enrich life at home (Eby et al., 2010). Precisely, enrichment happens when resources gained at work help in enriching life at home, either directly or indirectly. It enriches life at home by stimulating energy through fulfilling basic human needs.

Previously, Larson (2018) found a positive relationship between ingratiation toward coworkers and relatedness need satisfaction. Teoh et al. (2019) conducted research on hospitality sector employees, especially casino VIP rooms employees who usually ought to work under a great deal of stress. They found that adopting emotional regulation was positively related to relatedness need satisfaction of the employees. Based on the above notions of SDT and the COR theory, and in the light of the findings from the previous research, the current study posits the following hypotheses:

***H2a:*** *There is a positive relationship between workplace interactional demands and relatedness.*

***H2b:*** *There is a positive relationship between relatedness and work-family enrichment.*

***H2c:*** *Relatedness mediates the relationship between workplace interactional demands and work-family enrichment.*

### Workplace Interactional Demands, Personal Accomplishment and Work-Family Enrichment

Work-family enrichment stems from the role accumulation theory (Sieber, 1974). The fundamental notion of role accumulation theory in explaining the concept of work-family enrichment is that energetic involvement in family and employment domains together is accompanied by access to experiences and opportunities that lead to personal accomplishment or individual fulfillment. Another research conducted by Voydanoff (2008) also stressed the importance of psychological and structural assets as pertinent resources in enhancing work-family enrichment. Ecological systems approach was used by Voydanoff (2008) in order to present a conceptual framework that observed resources and demands across different life domains.

Resources are essential individual assets that have the tendency to shape work-family enrichment through improvement in the performance of family and employment roles. It enhances individual well-being through the transfer of skills and psychological development (Grzywacz et al., 2002; Voydanoff, 2004). The COR theory (Hobfoll, 2001) offers indisputable empirical evidence for the identification of different forms of resources that include energy resources, condition resources, and support resources. These resources help the employees to acquire time, knowledge, and money; and, hence, provide them with the opportunities of enrichment across different domains of life. Condition resources offer job prestige and generate feelings of accomplishment in the employees, which can serve as essential antecedents to work-family enrichment. Support resources also enhance enrichment through the preservation of conditions and energy resources. Professional jobs that typically require higher levels of commitment on the part of employees usually demand long work hours as compared to clerical or production jobs (Jacobs and Gerson, 2004). Females and working mothers usually prefer such jobs as they provide them with a sense of achievement and personal accomplishment (Damaske and Gerson, 2008). These feelings enhance the resource reservoir of employees and can be transmitted into family lives via psychological spillover of feelings, emotions and sensations (Voydanoff, 2004). Previous research has found emotional labor to be related to personal accomplishment. Bhowmick and Mulla (2016) and Aziz et al. (2018) found deep acting emotional labor to be positively related to personal accomplishment. Similarly, Nair (2019) found that personal accomplishment mediated the relationship between strategies of emotional labor and teaching satisfaction among professional college teachers. The above literature states that workplace interactions fulfill the human needs for accomplishment and females (nurses) prefer such jobs as they fulfill their accomplishment need, so the following hypotheses are proposed:

***H3a:*** *There is a positive relationship between workplace interactional demands and personal accomplishment.*

***H3b:*** *There is a positive relationship between personal accomplishment and work-family enrichment.*

***H3c:*** *Personal accomplishment mediates the relationship between workplace interactional demands and work-family enrichment.*

**FIGURE 1:**
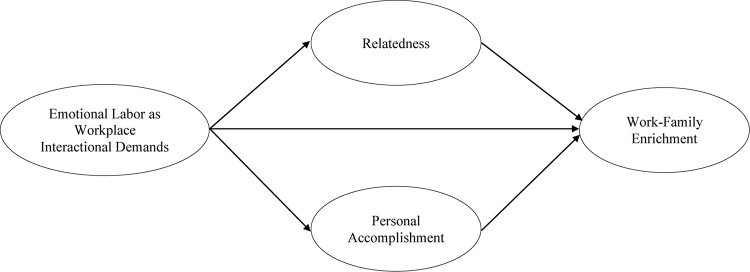
Conceptual framework.

The conceptual framework is presented in Figure 1.

## Methodology

### Sample and Procedure

The target population of the study was nurses of Public and private sector hospitals in the three biggest regions of the province of Punjab, Pakistan, i.e., Lahore, Faisalabad, and Multan. The data were collected through self-administered questionnaires. In order to qualify for inclusion in the survey, a nurse must have been working for the last 5 years and must be married. To mitigate the non-response bias, we included screening questions regarding marital status and tenure of the respondents.

A poor survey construction and targeting skill from the questionnaire are two major reasons for non-response bias. Our questionnaire did not suffer from poor survey construction as we extended full assurance of the anonymity to the respondents. We obtained informed consent from the participants; moreover, we did not include any sensitive questions in the survey. Targeting was also not a problem as we included clear screening questions in the survey in order to ensure that the survey reached only to the relevant respondents. Therefore, the demographic profile of non-respondents was not substantially different from the respondents. Non-response bias, thus, did not pose any significant threat to the validity of our findings.

We used convenience sampling methodology, which is widely used in quantitative studies (Etikan et al., 2016). Convenience sampling is a type of non-probability sampling in which those population members are recruited who are conveniently available to participate in the study. Köhler et al. (2017) suggested that in the majority of management research areas, studies are often based on convenience samples. We believe that our theories are adequately applicable to the sample of our study; we are, therefore, optimistic about the generalizability of our findings as proposed by Highhouse and Gillespie (2009).

A total of 550 questionnaires were distributed, out of which 423 were received back. Out of them, 45 were partly filled, and 14 were blank; therefore, our final sample size was 364 (response rate 66%). All the respondents were married female nurses working in different hospitals of Lahore, Faisalabad and Multan. More than half of the nurses were having a graduation degree (56%). They were holding different designations, and the majority of them were between 21–30 years of age (56.6%). The detailed demographic profile of the respondents is given in Table 1.

**TABLE 1:** Demographic characteristics of respondents.

	***n***	**Percentage**
**Organization**		
Government General Hospital Faisalabad	27	7.4
Faisal Hospital	27	7.4
Allied Hospital	49	13.5
Aziz Fatimah Hospital	26	7.1
Mian Muhammad Trust Hospital	14	3.8
Nawaz Medicare	3	0.8
Khadija Mahmood Trust Hospital	10	2.7
Omar Hospital & Cardiac Centre	8	2.2
Jinnah Hospital, Lahore	13	3.6
Services Hospital, Lahore	31	8.5
Mayo Hospital, Lahore	28	7.7
Lahore General Hospital	12	3.3
Punjab Institute of Cardiology, Lahore	23	6.3
Shalamar Hospital, Lahore	15	4.1
Sir Ganga Ram Hospital, Lahore	37	10.2
Nishtar Hospital, Multan	13	3.6
Children Hospital, Multan	12	3.3
Railway Hospital Multan	10	2.7
Government Mother and Childcare Hospital, Multan	6	1.6
**Qualification**		
Undergraduate	67	18.4
Graduate	204	56.0
Master’s Degree	58	15.9
Others	35	9.6
**Designation**		
General Nurse	114	31.3
Nurse-Midwife	43	11.8
Charge Nurse	121	33.2
Staff Nurse	84	23.1
Clinical Nurse	1	0.3
Dispensary Nurse	1	0.3
**Age**		
Below 20 Years	24	6.6
21–30 Years	206	56.6
31–40 Years	126	34.6
Above 40 Years	8	2.2
Total	364	

### Measures

All the variables of the current study were measured on a 7-point Likert scale. The detail regarding items and the scale anchors used for each variable is provided below:

#### Workplace Interactional Demands

We measured workplace interactional demands/occupational interactional requirements by using six items from Bhave and Lefter (2018) who used eight items from the O^∗^NET scale. Bhave and Lefter (2018) used eight items following the study of Glomb et al. (2004), who have conducted principal components analysis of job demand characteristics and calculated the factor loadings of all the activities loading on “emotional labor demands” factor. They retained the items that had factor loadings above 0.40. Next, in order to assess the appropriateness of the scale in the local context, the lead author conducted interviews with health administrators from the biggest hospital each from Faisalabad, Lahore, and Multan. In consultation with the health administrators, we removed following two items from the survey: “Contact with others,” and “Dealing with physically aggressive people.” As these items were found to be redundant and overlapping.

The sample items were included from two different domains, i.e., “Generalized Work Activities” domain, and “Work Context” domain. A sample item is “how often your job requires providing personal assistance, medical attention, emotional support, or other personal care to others such as co-workers, customers, or patients.” The scale items were assessed on a 7-point Likert scale ranging from “1 = Never to 7 = Daily.”

#### Relatedness

The variable of relatedness was measured by an 8-items scale developed by Van den Broeck et al. (2010). A sample item is “I really feel connected with other people at my job.” The scale items were assessed on a 7-point Likert scale ranging from “1 = strongly disagree to 7 = strongly agree.”

#### Personal Accomplishment

Personal accomplishment items were taken from personal accomplishment subscale that is part of the 22-item Maslach Burnout Inventory (MBI) (Maslach et al., 1986, 1996). The personal accomplishment subscale contains eight items that describe beliefs of competence and successful achievement at work, e.g., “I feel positively influencing people’s lives.” Each of the eight items asked respondents to describe their feelings on a 7-point Likert scale, ranging from “1 = never having those feelings to 7 = having those feelings daily.”

#### Work-Family Enrichment

This variable was measured by a 9-items scale developed by Carlson et al. (2006). The scale items include, “My involvement in my work puts me in a good mood and this helps me be a better family member.” The items were assessed on a 7-point Likert scale ranging from “1 = strongly disagree to 7 = strongly agree.”

### Common Method Bias

This study employed several procedural and analytical remedies to mitigate the potential threat of common method bias (CMB), which may arise because of the use of cross-sectional and single-source data. The anonymity of the respondents was ensured, and the respondents were assured that the confidentiality of their responses would be conserved at all levels. They were, thus, instructed to answer the questions as honestly as possible. Moreover, written informed consent was obtained from the respondents before administrating the survey. These steps served to reduce the risk of social desirability bias (Podsakoff et al., 2003). Some questions were reverse-coded. Furthermore, the independent variable and dependent variable were placed at separate positions in the questionnaire. This step was taken because placing IV and DV closely on the questionnaire could arouse retrieval of information from the respondents’ memory by providing common contextual cues, and hence, the correlation between IV and DV might be biased (Sudman et al., 1996; Podsakoff et al., 2003; Malhotra et al., 2006). To statistically assess the possibility of CMB, Harman’s single-factor test was executed. The single factor in an unrotated solution explained only 28.37% of the variance which is well below the acceptable threshold. Thus, it can be concluded that CMB was not a potential threat to the data.

### Data Analysis

Keeping in view the theoretical orientation of study constructs and relatively lesser development in theory, a sophisticated multivariate data analysis method was required (Hair et al., 2014). Due to this, variance-based structural equation modeling (PLS-SEM) has been used (Hair et al., 2011). For this purpose, SmartPLS 3.2.9 was used (Ringle et al., 2015).

### Assessment of the Measurement Model

The data were analyzed through measurement and structural models. A reflective measurement model was established while considering the relationships among study constructs and their indicators (Hair et al., 2016). The measurement model was assessed on the basis of reliability and validity (Table 2). Construct reliability was assessed through Cronbach’s Alpha, rho-A, and composite reliability (CR) (Bacon et al., 1995). All the values of alpha coefficients were above threshold; similarly, the values of CR and rho-A were also above the cutoff value, i.e., >0.70 (Hair et al., 2013) confirming the reliability of measurement model. On the other hand, validity was assessed on the basis of convergent and discriminant validity. Convergent validity is used to identify how an indicator is positively correlated with other indicators under the same umbrella of the theoretical framework (Chin, 2010). In the case of the reflective measurement model, it is assessed on the basis of outer loadings (Hulland, 1999; Mela and Kopalle, 2002; Chin, 2010) and average variance extracted (AVE) (Hair et al., 2013, Hair et al., 2016). In the first attempt, the indicators with lower outer loading values, i.e., below 0.708, were checked. Indicator EL-3 from workplace interactional demands was dropped due to poor outer loading while EL-1 and EL-5 were retained despite having slightly low outer loading values. Three items from relatedness were deleted because of low factor loadings. Similarly, some items form personal accomplishment and relatedness were also found with slightly lower outer loadings but these were retained because AVE of their respective constructs was above the threshold value, i.e., 0.50. Hence, all the values of AVE and outer loadings were within the acceptable range. However, in the case of workplace interactional demands, AVE was observed slightly below the cutoff value, i.e., 0.48, but such values are also acceptable (Malhotra et al., 2006).

**TABLE 2:** Indicator reliability, VIF, alpha, rho-A, CR, and AVE.

**Constructs**	**Indicator**	**Indicator Reliability**	**VIF**	**Alpha**	**rho-A**	**Composite Reliability**	**AVE**
Workplace Interactional Demands	EL1	0.673	1.268	0.85	0.85	0.89	0.494
	EL2	0.729	1.489				
	EL4	0.718	1.416				
	EL5	0.631	1.234				
	EL6	0.704	1.347				
Personal Accomplishment	PA1	0.717	1.700	0.75	0.76	0.83	0.503
	PA2	0.685	1.551				
	PA3	0.689	1.621				
	PA4	0.736	1.741				
	PA5	0.698	1.645				
	PA6	0.684	1.548				
	PA7	0.741	1.784				
	PA8	0.672	1.473				
Relatedness	RL1	0.743	1.428	0.92	0.92	0.93	0.601
	RL2	0.766	1.500				
	RL4	0.702	1.389				
	RL5	0.685	1.351				
	RL8	0.645	1.253				
Work-Family Enrichment	WFE1	0.740	1.969	0.73	0.73	0.82	0.478
	WFE2	0.790	2.368				
	WFE3	0.735	1.865				
	WFE4	0.760	2.032				
	WFE5	0.785	2.261				
	WFE6	0.800	2.455				
	WFE7	0.772	2.285				
	WFE8	0.793	2.253				
	WFE9	0.801	2.405				

*All factor loadings are significant at 0.05 level.*

Discriminant validity (see Table 3) was assessed through Fornell and Larcker (1981) criteria and HTMT (Hair et al., 2016). In the case of Fornell and Larcker (1981) criteria, square root of AVE of each latent variable was higher than the correlations among latent variables (Chin, 2010; Hair et al., 2011), confirming discriminant validity. In addition to this, HTMT values were less than the threshold value of 0.85 confirming the discriminant validity of the model (Henseler et al., 2015).

**TABLE 3:** Fornell–Larcker criterion, HTMT, coefficient of determination and predictive relevance.

	**Construct**	**1**	**2**	**3**	**4**	***R*-square**	***R*-square adjusted**	***Q*-square**
1	Personal Accomplishment	**0.703**	0.728	0.633	0.620	0.24	0.23	0.11
2	Relatedness	0.590	**0.710**	0.653	0.546	0.16	0.16	0.07
3	Work-Family Enrichment	0.565	0.545	**0.776**	0.501	0.40	0.39	0.22
4	Workplace Interactional Demands	0.490	0.408	0.410	**0.692**	–	–	–

*Values in bold and underlined are square root of AVE of respective constructs, while values above the bold and underlined indicate HTMT. All correlations were significant at 0.05 level.*

### Assessment of Structural Model

The structural model was assessed on the basis of VIF values (Table 2), coefficient of determination (Level of *R*^2^) (Table 3) also known as a measure of predictive accuracy, and the predictive relevance *Q*^2^ (Table 3) (Hair et al., 2013). Hence, in the first instance, values pertaining to multicollinearity (VIF) were assessed (Mela and Kopalle, 2002) which indicated best parameter estimation because all the values of indicators were below cutoff value of +5.0 (Hair et al., 2013). Next, the coefficient of determination (*R*^2^) was assessed to determine the percent of change in endogenous construct due to the exogenous construct(s) (Table 3). Twenty-four percent change in personal accomplishment and 16% change in relatedness was observed due to workplace interactional demands while combined 40% change was observed in work-family enrichment (endogenous construct) due to exogenous constructs of this study. In the case of effect size (*f*^2,^) near small effect sizes were observed for work-family enrichment due to personal accomplishment (0.101) and relatedness (0.096). Near medium effect sizes were observed for personal accomplishment (0.316) and relatedness (0.200) due to workplace interactional demands, and the small effect size was observed for work-family enrichment (0.020). Finally, predictive relevance was measured through *Q*^2^ (Table 3) and all the values of *Q*^2^ were found larger than 0.01 indicating the predictive relevance of reflective endogenous latent variables (Stone, 1974; Geisser, 1975).

### Hypotheses Testing

**FIGURE 2:**
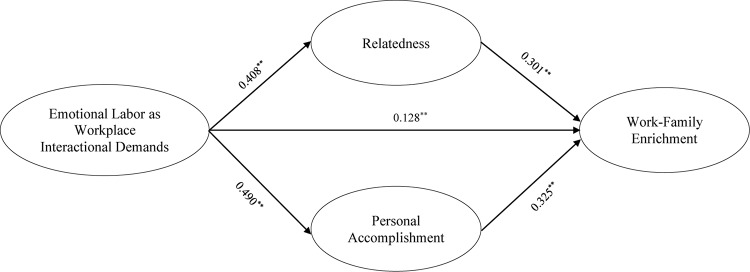
Path estimates.

Next, we proceed toward hypotheses testing. These results are presented in Table 4, and path estimates are elaborated in Figure 2. The results of the structural model indicated that workplace interactional demands was positively related to work-family enrichment (β = 0.128, *p* < 0.01). Thus, H1 was supported. Workplace interactional demands was positively related to relatedness as well as to personal accomplishment (β = 0.408 and 0.490, respectively, *p* < 0.01). Therefore, H2a and H3a were supported. Both mediators, i.e., relatedness (β = 0.301, *p* < 0.01) and personal accomplishment (β = 0.325, *p* < 0.01) were positively related to work-family enrichment. H2b and H3b were, therefore, supported.

**TABLE 4:** Hypotheses testing.

**Hypotheses**		**Beta**	***t***	***p***	**Status**
H1	Workplace Interactional Demands → Work-Family Enrichment	0.128	2.938	0.00	Supported
H2a	Workplace Interactional Demands → Relatedness	0.408	8.426	0.00	Supported
H2b	Relatedness → Work-Family Enrichment	0.301	4.771	0.00	Supported
H3a	Workplace Interactional Demands → Personal Accomplishment	0.490	10.987	0.00	Supported
H3b	Personal Accomplishment → Work-Family Enrichment	0.325	4.693	0.00	Supported

**Hypotheses**		**Indirect Effect**	**Total Effect**	**VAF**	**Status**

H2c	Workplace Interactional Demands → Relatedness → Work-Family Enrichment	0.123	0.410	30%	Supported
H3c	Workplace Interactional Demands → Personal Accomplishment → Work-Family Enrichment	0.159	0.410	39%	Supported

While testing mediating effects, we followed Preacher and Hayes (2008) and used bootstrap sampling distribution of the indirect effect (Preacher and Hayes, 2008; Hair et al., 2014). Both indirect effects were found to be positive and significant. The indirect effect of workplace interactional demands on work-family enrichment through relatedness can be obtained by multiplying *path a* (path from IV to mediator) with *path b* (path from mediator to DV). This indirect effect was 0.123 (0.408^∗^0.301 = 0.123, *p* < 0.01). Similarly, the indirect effect through personal accomplishment was 0.159 (0.490^∗^0.325 = 0.159, *p* < 0.01).

Mediation was assessed by calculating the variance accounted for (VAF). VAF determines the size of the indirect effect in relation to the total effect (direct effect + indirect effect). Here the value of VAF was 30% for relatedness, assuming that relatedness partially mediated the association between workplace interactional demands and work-family enrichment. Similarly, VAF was 39% for personal accomplishment, assuming that personal accomplishment partially mediated the association between workplace interactional demands and work-family enrichment.

## Discussion

The findings support the first hypothesis, which proposed that there is a positive association between workplace interactional demands and work-family enrichment. Previous studies have only reported an indirect association between interactional demands and enrichment through vitality (e.g., Bhave and Lefter, 2018). The current study supports a direct positive relationship between interactional demands and enrichment. This is consistent with previous studies that have shown a positive relationship of deep acting emotional labor with family quality (e.g., Liu et al., 2018) and work-family enrichment (e.g., Wu et al., 2020). Nurses, when interact with the patients and help them, gain resources. Personal resources are valuable in their own right and can serve to enhance the richness of family life.

In hypothesis 2a, we have proposed that there is a positive relationship between interactional demands and relatedness. The psychological need for relatedness relates to the individual’s inherent tendency to feel connected to other individuals or be a team member or group member to be loved and to be cared for Baumeister and Leary (1995). Workplace interactions satisfy the basic human need for relatedness. Furthermore, other development approaches, such as attachment theory (Bowlby, 1969) also proposes that human beings have a natural tendency to integrate themselves in social relationships and networks and get benefits from being loved and cared for. This theory is also consistent with other concepts of organizational psychology like loneliness felt at the workplace (Wright et al., 2006), and social support employees get at the workplace (Viswesvaran et al., 1999). These relationships enhance the energy reservoirs of service sector employees.

In hypothesis 2b, we have stated that there exists a positive relationship between relatedness and work-family enrichment. Previous studies have reported that the satisfaction of the basic psychological needs of humans results in the enhancement of the well-being of employees (Chirkov et al., 2003; Deci and Ryan, 2008). Moreover, Hanson et al. (2006) have stated that enrichment occurs when resources gained during the performance of one role directly/indirectly enhance the performance of the other roles. Therefore, we also interpret that enrichment exercises a positive impact on the occupational and mental health of employees. These findings are consistent with past studies (McNall et al., 2010). More specifically, it is interpreted that resources gained in one role result in employee development (Crouter, 1984; Haas, 1999; Edwards and Rothbard, 2000). It also has positive implications in the field of employee development as a healthy and satisfied worker is a productive worker, and such a workforce paves avenues for organizational development as well.

In hypothesis 2c, we have proposed that relatedness mediates the relationship between workplace interactional demands and work-family enrichment. As mentioned above, work-family enrichment may be defined as the extent to which experiences in one role improve the quality of life in the other role (Greenhaus and Powell, 2006). An employee working in an organization is not only the organizational employee but also have other roles to perform like those of family member and the member of the society. Employee’s work and family life can be a source of enrichment for the other domain through the resources gained during the performance in either role of life (De Klerk et al., 2014). These resources are gained through basic psychological needs satisfaction. Therefore, we propose that workplace interactional demands generate valuable subjective resources that can activate employee’s resources of relatedness. The need for relatedness is very important, and it must be satisfied as it is linked to the well-being of employees. The primary task of nurses is to take care of the patients, and by doing so, they might fulfill their need for relatedness. These resources, as a result of satisfaction of basic needs, are helpful beyond the work domain and can be transferred to the home domain, which in turn can enhance work-home enrichment (ten Brummelhuis and Bakker, 2012). Furthermore, employees’ quality of life at home is enriched through these resources, and vice versa (Powell and Greenhaus, 2006; Eby et al., 2010; Bhave and Lefter, 2018).

Our findings also support hypothesis 3a, which states that there exists a positive relationship between workplace interactional demands and personal accomplishment. Working in the service occupations that involve frequent interactions with the beneficiaries of their work, particularly in jobs that are accompanied by a high degree of altruism which accompanies helping others in need, enhances the well-being of the employees (Yurcu et al., 2015). Human beings from the past until now have the desire to sense and feel satisfied and contented, and these good feelings enhance their well-being. Therefore, it results in personal accomplishment for employees by showing kindness toward others. It is a proven phenomenon that individuals who possess higher levels of subjective well-being are more effective in problem-solving, exhibit kindness or altruism, and have more stress-resistant capabilities (Veenhoven, 1991).

Our findings also confirm hypothesis 3b, which states that there is a positive relationship between personal accomplishment and work-family enrichment — personal accomplishment results in the gain of resources that can be transferred from work domain to home domain. Personal accomplishment occurs when an individual is competent and successfully achieves the goals of his or her job (Maslach et al., 1996). Interactions with others at the workplace especially acts of kindness that can be life-saving for others, provide the employees with a sense of achievement and competence. This provides employees both with motivational (Grant, 2007) and physiological (Heaphy and Dutton, 2008) resources that not only energize them but also enhance their well-being (Grant, 2008; Grant and Parker, 2009). We have also confirmed the notions of the role accumulation theory, which states that engaging in multiple roles is beneficial for the mental, physical, and relational health of the individuals (Barnett and Hyde, 2001). Resources gained by performing job roles provide employees with a sense of personal accomplishment (Janssen et al., 1999).

The findings of our research also confirm hypothesis 3c, which states that personal accomplishment mediates the relationship between workplace interactional demands and work-family enrichment. Past researchers have reported that workplace interactional demands result in employee satisfaction and help to protect their self-identity (Curley and Royle, 2013; Humphrey et al., 2015), and result in personal accomplishment (Brotheridge and Grandey, 2002). The previous studies in this regard contend that the active involvement of an individual in multiple domains of family and work provides them with the opportunities to enhance their levels of individual fulfillment or personal accomplishment. This can be fruitful for healthy family life.

As we all know that time is not infinite, instead it is a finite resource that cannot be stored (Halbesleben et al., 2014); that is why time allocation is very important and it lays emphasis on family routines. The nurses have to work for long hours and must have adequate resources at their disposal to be consumed in the family. So, the current study essentially confirms that resources gained in the work domain while engaging in workplace interactions result in work-family enrichment.

The findings of our research are in contrast with the old conventional notion that emotional labor is resource depleting or harmful for the employees working in service occupations and challenges the established assumption that emotional labor or interactional demands are resource depleting (resource-draining) and are a cause of burnout (Hochschild, 1983; Grandey et al., 2015). With the expansion of the service industry, there is a dire need to highlight the positive aspects of service occupations. Modern research has shifted the focus toward the other side of emotional labor. Our study has contributed to the positive side of interactive jobs by establishing direct as well as the indirect positive association of interactive jobs and enrichment.

### Theoretical Implications

The current research contributes to the empirical literature on emotional labor by endorsing a positive direct relationship between workplace interactional demands and work-family enrichment. This study has important contributions toward emerging research on emotional labor. It has taken the studies of Bhave and Lefter (2018) a step further. They have established the indirect effect of interactional demands on work-family enrichment. The current research has examined the direct effects of interactional demands as well as indirect effects through personal accomplishment and relatedness on work-family enrichment. The structure of work is dependent upon occupations; furthermore, they also influence how work is performed, and how employees experience work. Nursing is one of the occupations that have high values of altruism and is accompanied by interactional demands that are highly important. The current research might help enhance the work-family enrichment of nurses. As nurses have to work for extended hours and they must replenish their resources at work rather than looking for breaks. Summing it up, we can say that the current research is beneficial for researchers as well as for policymakers that increasing the meaningfulness of jobs in which the basic needs of employees are met is resource replenishing. Our research focused on employee recovery at work and recovery resources, which are very important for the optimal functioning of individuals across life domains.

This research extends the COR theory in a meaningful way by showing that resources gained through personal interactions at the workplace are valuable for the employees. Thus, employees tend to generate and conserve these resources. These resources can be transferred from the work domain to the home domain and serve to enrich the family life. Moreover, SDT proposes that intrinsic motivation could be drawn from the fulfillment of the need for autonomy, relatedness and competence. Drawing on SDT, we show that meaningful workplace interactions primarily serve to fulfill the innate human needs. When employees indulge in workplace interactions, it satisfies the need for relatedness that emphasizes on their inherent tendency to make connections. These connections are satisfying and energize employees as it triggers their natural motivational mechanisms. This energy can be a valuable resource and can be transferred to the home domain. The fulfillment of these needs spills over from the work domain to the family domain and the individuals get enrichment in their family lives.

### Practical Implications

Nursing staff experience many problems like extensive workload, low pay scales, and feelings of undervaluation, insufficient compensation, workplace violence, long working hours, and most important of them are the workplace health hazards. It is imperative for their employers to do their homework not only to make their jobs meaningful but also to focus on their enrichment. Work-family enrichment is significant for employees in the service sector.

Firstly, work–home enrichment is crucial for employee’s next day work engagement. The nursing staff has to work for long hours, and recovery at work itself is vital for the health sector staff as they do not have ample time at their disposal to recover resources for other domains of life. Earlier research has stated that if the employees’ resources are recovered at home, they feel themselves energized, and it is very crucial for their next day work engagement. The current study states that workplace interactions are energizing and they help in the enrichment of family life and vice versa. Thus, our study is constructive for those employers who are in search of healthy, committed and productive workforce.

In conclusion, our research removes all the myths about workplace interactions and establishes that these should not be considered as unfavorable instead they must be considered beneficial for both the employers as well as employees.

### Limitations and Directions for Future Research

No study is without limitations, and this study is not an exception. First of all, we have taken only female workers working as nurses that are married and have children. We have not taken into account single nurses or nurses whose dependents are elderly persons. Secondly, we have relied only on the opinions elicited from the nurses themselves regarding work-home enrichment; we have not sought the opinions of other family members (spouse and other family members). The findings could be interpreted more realistically if we take into account the opinions of spouse and other dependent family members of the nurses. Thirdly, we have not taken into account alternative ways by which the employees can recover their resources. These can be recovered in alternative ways like engaging in leisure activities or vacations and breaks introduced in work for the purpose of recovery. Furthermore, we have recruited nurses to participate in the study as our sample, and there is a greater degree of direct interaction of nurses with the beneficiaries of their work. It may be the reason for the stronger relationship between interactional demands and work-family enrichment. Other service occupations with a lesser degree of interaction with the customers or beneficiaries might not reveal the same results.

Future researchers might introduce moderators in the relationship between interactional demands and enrichment. Moreover, we have only taken into account the interactional demands as resource replenishing centers for the employees; future research might introduce other mechanisms that can be resource replenishing to the employees. It may also be fruitful to check the relationship between resource-generating and resource-draining background mechanisms in the relationship between conflict and enrichment. The conceptual foundations on which the research can be carried out can be taken from the proposition that both resource-draining and resource-generating variables can have an impact upon work engagement and employee burnout (Bakker and Demerouti, 2008, 2017). So, future researchers can also study the impact of interactional demands on employees’ next day work engagement that can be very fruitful for organizational health and development.

## Disclosure

There is no conflict of interest in this study, and this study was carried out only for educational purposes. This study is based on perceptions of respondents, and no experimental or other research design was followed. However, we had obtained written informed consent from every respondent at the time when questionnaires were get filled. Moreover, respondents were not asked to disclose their names or any other information as a matter of confidentiality and their identity is anonymous.

## Data Availability Statement

The datasets generated for this study are available on request to the corresponding author.

## Ethics Statement

The studies involving human participants were reviewed and approved by the Ethics Committee of Government College University Faisalabad, Pakistan. The patients/participants provided their written informed consent to participate in this study.

## Author Contributions

SSol, MA, and SSal: definition of the research objectives, models, hypotheses, and principal manuscript crafting. RS, MB, and SSaq: the provision of materials (i.e., questionnaires). SSol, MA, and MB: data collection. SSal, RS, and SSaq: data analysis plan and data analysis. MA, RS, and SSal: manuscript revision and proofreading. SSol, MA, RS, MB, SSal, and SSaq: final approval.

## Conflict of Interest

The authors declare that the research was conducted in the absence of any commercial or financial relationships that could be construed as a potential conflict of interest.
